# MDR-TB Outbreak among HIV-Negative Tunisian Patients followed during 11 Years

**DOI:** 10.1371/journal.pone.0153983

**Published:** 2016-04-28

**Authors:** Naira Dekhil, Nedra Meftahi, Besma Mhenni, Saloua Ben Fraj, Raja Haltiti, Sameh Belhaj, Helmi Mardassi

**Affiliations:** 1 Unit of Typing & Genetics of Mycobacteria, Laboratory of Molecular Microbiology, Vaccinology, and Biotechnology Development, Institut Pasteur de Tunis, Université Tunis El Manar, Tunis, Tunisia; 2 Hôpital Régional de Menzel-Bourguiba, Menzel Bourguiba, Tunisia; St. Petersburg Pasteur Institute, RUSSIAN FEDERATION

## Abstract

**Background:**

Multidrug-resistant tuberculosis (MDR-TB) outbreaks that evolve, from the outset, in a context strictly negative for HIV infection deserve special consideration since they reflect the true intrinsic epidemic potential of the causative strain. To our knowledge, the long-term evolution of such exceptional outbreaks and the treatment outcomes for the involved patients has never been reported hitherto. Here we provide a thorough description, over an 11-year period, of an MDR-TB outbreak that emerged and expanded in an HIV-negative context, Northern Tunisia.

**Methodology/Principal Findings:**

From October 2001 to June 2011, the MDR-TB outbreak involved 48 HIV-negative individuals that are mainly young (mean age 31.09 yrs; 89.6% male) and noninstitutionalized. Drug susceptibility testing coupled to mutational analysis revealed that initial transmission involved an isolate that was simultaneously resistant to isoniazid, rifampicin, ethambutol, and streptomycin. The causative Haarlem3-ST50 outbreak strain expanded mainly as an 11-banded IS*6110* RFLP profile (77.1%), from which a 12-banded subclone evolved. After undergoing a 2-year treatment with second-line drugs, 22 (45.8%) patients were cured and 3 (6.2%) completed treatment, thus yielding an overall treatment success rate of 52.1%. Among the patients that experienced unfavorable treatment outcomes, 10 (20.8%) failed treatment, 3 (6.2%) were lost to follow-up, 5 (10.4%) died, and 5 (10.4%) could not be evaluated. Poor adherence to treatment was found to be the main independent predictor of unfavorable outcomes (HR: 9.15; 95% CI 1.72–48.73; *P* = 0.014). Intriguingly, the evolved 12-banded subclone proved significantly associated with unfavorable outcomes (HR: 4.90; 95% CI 1.04–23.04, *P* = 0.044). High rate of fatality and relapse was further demonstrated at the long-term, since 70% of those whose treatment failed have died, and 24% among those deemed successfully treated have relapsed.

**Conclusions/Significance:**

Taken together, the data obtained in this study indicate that MDR-TB clinical isolates could become fit enough to cause large and severe outbreaks in an HIV-negative context. Such MDR-TB outbreaks are characterized by low treatment success rates and could evolve towards increased severity, thus calling for early detection of cases and the necessity to raise the bar of surveillance throughout and beyond the treatment period.

## Introduction

Multidrug-resistant tuberculosis (MDR-TB) is defined as TB that is resistant to at least isoniazid and rifampicin, the two most effective front-line anti-tubercular drugs. Recent global estimates have uncovered the worrying trends of MDR-TB [1]. Indeed, among the 480 000 cases of MDR-TB recorded worldwide in 2014, 190 000 have died [2]. Not only is MDR-TB associated with high death rates, it is also difficult to manage and very expensive to treat, compared to drug-susceptible TB [1].

Basically, MDR-TB clinical strains are assumed to be transmission-deficient, and unlikely to spread in the community due to their reduced fitness. These strains bear multiple mutations associated with important biological functions, such as rifampicin resistance mutations within the β subunit gene of the RNA polymerase (*rpoB*) [3]. Drug resistance-conferring mutations impart a fitness cost to the strain that is reflected in its reduced growth, inefficient transmission, and/or changes in its relative virulence [4]. Studies carried out *in vitro* clearly showed the growth defect of MDR *M*. *tuberculosis* clinical isolates. Furthermore, aside from a few cases [5–7], the majority of MDR-TB outbreaks described thus far have been found to be associated with HIV-positive patients, thus reinforcing the notion that MDR-TB strains are poorly fit and could not successfully expand in the general community [8–10]. However, it has been shown that the fitness levels of some drug-resistant *M*. *tuberculosis* clinical isolates is similar to those of wild-type bacilli [8,11,12], suggesting that compensatory adaptive evolution could have operated to generate more fit MDR-TB clinical strains. This has been supported by a pioneering study, which provided strong evidence for the role of compensatory evolution in the global epidemics of MDR-TB [13].

In Tunisia, a middle-income country of about 11 million inhabitants, a national TB programme (NTP) was implemented since 1959, which adhered tightly to the guidelines recommended by the World Health Organization. This NTP relies on a laboratory network that includes 62 peripheral and 9 central laboratories, ensuring a good nationwide coverage. TB control is based on systematic double BCG vaccination at birth and at entry to school, passive case detection with sputum microscopy, and prompt short-course multidrug therapy administered under direct supervision of all identified cases. Full implementation of the five major components of the Directly Observed Treatment, Short-course (DOTS) programme was achieved in 1999, and a significant decrease in TB incidence was observed between 1975 and 2002 (from 48.6 down to 18.9 per 100 000 inhabitants) [6]. In the last decade, TB incidence has registered a steady increase, reaching 33 per 100 000 inhabitants in 2014 [2]. MDR-TB burden remains very low in Tunisia; being estimated at about 0.8% and 12% among new and retreatment cases, respectively [2].

However, in 2001, we observed a sudden increase in the number of MDR-TB cases among TB patients presenting to the regional hospital of Menzel-Bourguiba, which deserves the whole region of Bizerte, northern Tunisia [6]. Between years 2001 to 2004, 20 HIV-negative and noninstitutionalized patients were diagnosed with MDR-TB, thus prompting a detailed epidemiological and molecular study. These MDR-TB cases were attributed to an outbreak involving a Haarlem3-ST50 *M*. *tuberculosis* isolate [6]. Here, we provide a thorough description of this MDR-TB outbreak over an 11-year period (2001–2011), which is a prerequisite for future detailed studies aiming at deciphering the molecular basis underlying its successful expansion.

## Materials and Methods

### Ethics statement

This study was approved by the Biomedical Ethics Committee of the Institut Pasteur de Tunis. The sputum samples used in this study have been forwarded to the Unit of Typing & Genetics of Mycobacteria of the Institut Pasteur de Tunis, as part of its routine diagnostic activity that is allowed by the Tunisian Ministry of Health, in the context of the National TB program. The data were analyzed anonymously and, in no case, could the identity of the patients be deduced or disclosed.

### Setting and patients

The MDR-TB outbreak described herein has emerged in the region of Bizerte, and was due to a strain belonging to the Haarlem3 genotype (ST50) [6]. Bizerte occupies an area of 3 685 km^2^ with 568 219 inhabitants, and an estimated TB incidence of 29 per 100 000. All patients suspected of having TB living in the region of Bizerte are referred to the regional hospital of Menzel-Bourguiba. In the period 2001–2011, all sputum samples from suspected TB patients presenting to this regional hospital were forwarded to the Unit of Typing & Genetics of Mycobacteria of the Institut Pasteur de Tunis. Therefore, our laboratory ensured a full coverage of TB cases from Bizerte, including the MDR-T Boutbreak.

Following the government regulations, all suspected TB patients submit three sputum samples for both acid-fast smear and mycobacterial culture. First-line anti-TB treatment is initiated as soon as a positive acid-fast bacillus (AFB) smear or culture is demonstrated. Patients with negative AFB smears are subjected to chemotherapy in case of clinical evidence of TB. TB patients are reported, registered, and interviewed to obtain detailed epidemiologic data.

Sputum samples from patients with culture-positive TB are collected for mycobacterial culture every month or two months, whenever possible. Drug susceptibility testing (DST) are performed systematically since 2001, and MDR cases are immediately subjected to second-line chemotherapy.

### Sputum samples processing and drug susceptibility testing

All sputum samples were decontaminated and liquefied according to the Petroff method (4% NaOH). The smears were examined by Ziehl–Neelsen staining. Sputum was graded according to the number of visible acid fast bacilli. The WHO/IUATLD four grading categories were adopted for positive smears: 1–9 AFB (actual number of AFB seen on whole slide), 1+ (10 to 99 AFB in 100 fields), 2+ (1 to 10 AFB per fields in at least 50 fields), 3+ (>10 AFB/field in 20 fields).

Primary isolation and culturing of *M*. *tuberculosis* isolates in Löwenstein-Jensen (LJ) medium were performed as described elsewhere [14]. DST was performed according to the proportion method on LJ medium. The drugs used and their critical concentrations were as follows: isoniazid (INH) (0.2 μg/mL), rifampicin (RIF) (40 μg/mL), streptomycin (STM) (4 μg/mL), ethambutol (EMB) (2 μg/mL) and pyrazinamide (PZA) (200 μg/mL). The criterion used for drug resistance was growth of 1% or more of the bacterial population on media containing the critical concentration of each drug.

### Course of anti-TB chemotherapy

All newly diagnosed TB patients received the standard first-line chemotherapy regimen adopted by the Tunisian National Tuberculosis Program. It consists of 2 months of isoniazid, rifampicin, pyrazinamide, and ethambutol, followed by 4 months of rifampicin and isoniazid. Once multidrug resistance is established, patients received a treatment consisting of ethambutol, pyrazinamide, amikacin, levofloxacin, and ethionamide for 6 months, followed by at least an 18-month administration of ethambutol, pyrazinamide, ofloxacin, and ethionamide. MDR-TB cases are requested to provide sputum every month during the first six months until conversion, and then every 2 months till the end of treatment. These standard regimens could be adjusted according to adverse drug effects.

### Data collection and case definitions

Case data were collected prospectively by the public health staff using a standardized questionnaire. Information was obtained on gender, age, address, close contacts, previous TB history and associated medical data, such as human immunodeficiency virus (HIV) infection, underlying diseases (diabetes mellitus, cancer, liver cirrhosis, end-stage renal disease). Chest radiography findings are recorded.

MDR-TB case definition was based on the WHO definitions and reporting framework for tuberculosis that was revised in 2013 and updated in December 2014 [15]. Briefly, new patients are those who have no history of prior TB treatment, or they have received less than one month of anti-TB drugs. Previously treated TB patients include those patients treated as new cases for more than one month who are smear or culture positive. The treatment outcomes of MDR-TB patients were reported according to the following six outcome categories: (i) “Cured”: treatment completed as recommended by the national policy without evidence of failure and three or more consecutive cultures taken at least 30 days apart are negative after the intensive treatment phase; (ii) “Treatment completed”: treatment completed as recommended by the national policy without evidence of failure but no record that three or more consecutive cultures taken at least 30 days apart are negative after the intensive phase; (iii) “Treatment failed”: Treatment terminated or need for permanent regimen change of at least two anti-TB drugs; (iv) “Died”: a patient who died for any reason during the course of treatment; (v) “Lost to follow-up”: a patient whose treatment was interrupted for 2 consecutive months or more; (vi) “Not evaluated”: A patient for whom no treatment outcome is assigned (it could be a transfer out case).

The outcome categories “cured” and “treatment completed” were further summarized as treatment success, whereas the outcomes “treatment failed”, “died”, “lost to follow-up, and “not evaluated” were deemed unsuccessful (unfavorable) treatment outcome.

### Molecular typing of *M*. *tuberculosis* clinical isolates

A loopful of a 3-week LJ culture of *M*. *tuberculosis* was resuspended in 500 μL ultrapure nuclease-free water (Amersham Biosciences). After a 30-min incubation of the mixture at 80°C, the heat-killed bacteria were centrifuged at 12 000 rpm for 5 min, and 5 μL of the supernatant were used for subsequent PCR amplifications, typing purposes, and mutational analyses.

Spoligotyping was performed using the standard protocol described by Kamerbeek et al.[16]. Spoligotyping patterns and their corresponding shared types (ST) were defined according to the definitions in the SITVITWEB database (http://www.pasteur-guadeloupe.fr:8081/SITVIT_ONLINE/). The 24-loci MIRU-VNTR (MIRU-VNTR24) typing was performed[17]. PCR products were analyzed on a 2% Metaphor agarose gel (BioWhittaker Molecular Applications, Rockland Maine) using a 100-bp DNA ladder (Invitrogen) as the size marker. The ethidium bromide-stained gel images were captured and the copy number of each locus was calculated by applying the corresponding conversion table available at the web site (http://www.miru-vntrplus.org).

Previously we showed that the outbreak strain evolved mainly as an 11-banded IS*6110*-RFLP profile, whereas some strains showed an additional IS*6110* band (12-banded IS*6110* RFLP profile) resulting from IS*6110* insertion between Rv1645c and Rv1646c [18]. To distinguish between the two IS*6110* RFLP profiles of the outbreak strains, we performed an insertion site-specific PCR using primers flanking IS*6110* insertion between Rv1645c and Rv1646c (1855675:1855676; coordinates relative to H37Rv genome)[18]. A PCR product with a size of 1.6 kb is indicative of a 12-banded outbreak strain, while an amplicon of 0.3 kb is characteristic of an 11-banded outbreak strain.

### Mutational analysis of drug resistance-conferring genes

For molecular detection of drug resistance, we used, whenever possible, the very first patient’s isolate or, alternatively, the very first isolate available to us. The following loci were amplified by PCR: *rpoB*, *katG*, *inhA*, *pncA*, *embB*, *rrs*, *rpsL*, *gidB*, *gyrA*, *tlyA*, and *eis*. The primer sequences and amplicon sizes are presented in S2 Table. Each 20μL PCR mixture contained 10μL of HotStar *Taq* master mix (Qiagen); 1 μL of the forward and 1 μL of the reverse 5μM primers; 0.5μL of dNTP 10 mM (Amersham Biosciences); 4μL of genomic DNA and 3.5μL of ultrapure nuclease-free water (Amersham Biosciences). PCR was carried out for 35 cycles (an initial denaturation step at 94°C for 5min, followed by denaturation at 94°C for 1min), annealing temperature for different genes at 60°C (except *rpoB* at 64°C) for 30 s and elongation at 72°C for 2 min, with a final elongation step at 72°C for 7 min. Amplification was performed on a Veriti 96-well Thermal cycler (Applied Biosystems) and amplified products were detected by 1% agarose gel electrophoresis. Sequencing reactions were performed on an ABI Prism 3110 genetic analyzer using BigDye^™^ Terminator chemistry (Applied Biosystems) according to the manufacturer’s instructions. Nucleotide and amino acid sequences of the amplified fragments were aligned with the corresponding sequences of the reference *M*. *tuberculosis* H37Rv strain from Tuberculist (http://tuberculist.epfl.ch). All detected mutations were compared to those included in the TB drug resistance mutation database (www.tbdreamdb.com) [19].

### Statistical analyses

Data were presented as number (%) unless otherwise indicated. Intergroup difference was calculated using independent-sample *t* test for continuous variables and the chi-square test or Fisher’s exact test for categorical variables, as appropriate. To evaluate the predictors for unfavorable outcomes, we compared selected clinical variables between the favorable outcome and the unfavorable outcome groups, using univariate analyses. Variables significantly associated with unfavorable outcomes in univariate analysis (*P*< 0.05) were subsequently used in a multiple logistic regression analysis. For this purpose, stepwise and backward selection procedures were performed to select variables to be maintained in the final model.

Kaplan–Meier survival curves for significant predictors of unfavorable outcomes were constructed and analyzed using the log-rank test.

Statistical analyses were performed using SPSS statistical software (version 19.0; SPSS Inc., Chicago, IL, USA). Two-sided *P*< 0.05 was considered statistically significant.

## Results

### Genotypic and epidemiological features of the Haarlem3 MDR-TB outbreak

Between October 2001 and June 2011, 48 culture-confirmed MDR-TB cases related to the previously described Haarlem3 MDR-TB outbreak [6]were diagnosed in the region of Bizerte, northern Tunisia. Assignment of the MDR *M*. *tuberculosis* isolates to the ongoing Haarlem3 outbreak was first achieved by molecular typing (spoligotyping and MIRU-VNTR24). All MDR-TB isolates displayed the spoligo-signature of the Haarlem3-ST50 genotype (absence of spacers 31 and 33 to 36) and showed nearly identical MIRU-VNTR24 profiles. Indeed, 46 isolates were indistinguishable, while the remaining two isolates, which were identical, differed at a single locus (S1 Table). Unambiguous assignment of the 48 MDR-TB isolates to the Haarlem3 outbreak and confirmation of their clonal nature was further demonstrated by the fact that they all displayed the very rare and outbreak-specific 3’ allele at MIRU locus 4 (S1 Table).

Aside from these 48 MDR Haarlem3 outbreak-associated isolates, 7 additional MDR strains could be detected among the 557 isolates recovered from the outbreak region from 2001 to 2011. Based on their distinct spoligopattern and mutational profiles of drug resistance genes, these MDR strains could not be linked to the Haarlem3 MDR outbreak (data not shown).

**Fig 1:**
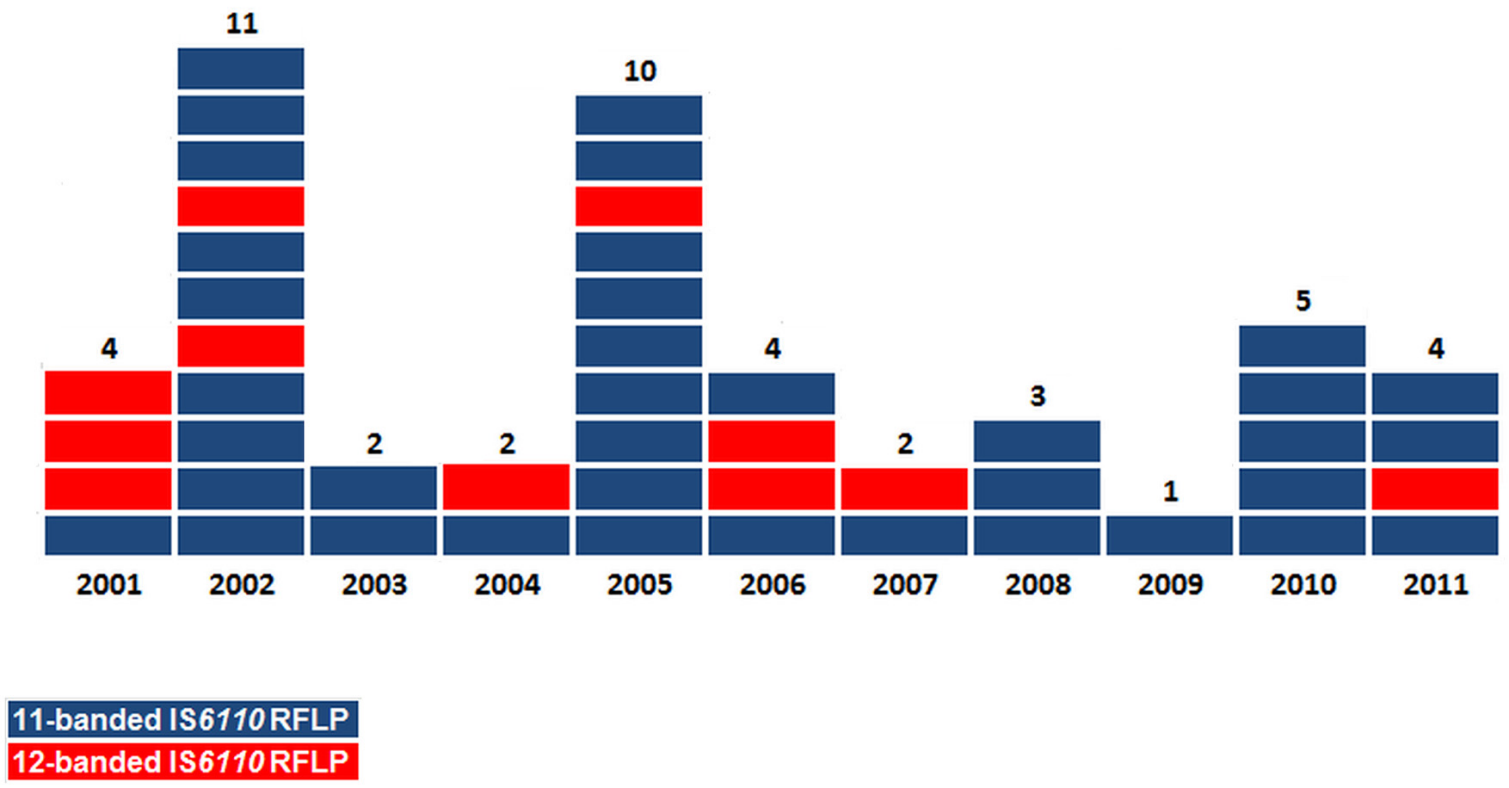
Temporal distribution of the 48 Haarlem3 MDR-TB outbreak cases, 2001–2011.

Using a PCR test targeting the insertion site corresponding to this additional IS*6110* band [18], we confirmed that the 11-banded strain was predominant among the 48 MDR-TB outbreak patients, accounting for 77.1% of cases (Table 1). Fig 1 shows the temporal distribution, throughout the whole study period (2001–2011), of the MDR-TB outbreak new cases, according to the IS*6110*RFLP profile of their corresponding isolates.

**Table 1:** Baseline demographic characteristics of the 48 MDR-TB outbreak-associated patients according to the IS*6110*-RFLP profile of their corresponding isolate.

	All patients n (%)	IS*6110* RFLP profile		*P* value
		11-banded	12-banded	
**N°**	48 (100%)	37 (77.1%)	11 (22.9)	
**Age mean (SD)**	31.09 (12.85)	32.06 (14.01)	28.09 (8.11)	0.380
**Male**	43 (89.6%)	33 (89.2%)	10 (90.9%)	1.000
**HIV-positive**	0 (00.0%)	0 (00.0%)	0 (0.00%)	-
Positive AFB smear prior to SLD*	20 (41.6%)	13 (35.1%)	7 (63.6%)	0.162
**PZA resistance**	35 (72.9%)	24 (64.9%)	11 (100%)	**0.023**
**Incarceration**	12 (25%)	10 (27%)	2 (18.1%)	0.694

*SLD: Second line drugs

Significant *P* values are shown in bold.

Patients involved in the Haarlem3 MDR-TB outbreak all originated from the region of Bizerte. They had a median age of 31.15 years (range 15–70 yrs), 43 (89.6%) of whom were male. All patients tested HIV-negative (Table 1). Apart from23 patients that could have been in close contact with known outbreak cases (11 household contacts and 12 incarcerated cases), no apparent epidemiological links could be traced for the remaining 25 MDR-TB cases. Twenty (41.6%) patients were smear-positive and 35 had been infected with a strain displaying simultaneous resistance to the four first line anti-tubercular drugs. With the exception of PZA resistance, which is significantly associated with the 12-banded profile (*P* = 0.044), there were no significant differences between patients infected with the 11- and 12-banded strains regarding age, sex, AFB smear and culture results, or incarceration status (Table 1).

### Phenotypic and molecular characterization of drug resistance

DST results revealed that all Haarlem3 outbreak-associated isolates were resistant to the four first-line drugs, isoniazid, rifampicin, ethambutol, and streptomycin (S1 Table). Consistent with this finding, all these isolates harbored the same drug resistance-conferring mutations S315T, S531L, M306I, and R47Win their *katG*, *rpoB*, *EmbB*, and *gidB* genes, respectively (S1 Table). In addition, all these isolates displayed the mutations *rpoB* V615M, *tlyA* A33G, *gyrA* S95T, and *gyrA* E21Q (S1 Table), which further confirmed their clonal expansion.

Resistance to pyrazinamide could only be detected in 35 (72.9%) cases involving diverse mutations in the *pncA* gene: A-11C, T-8C, L4W, Q10H, G10P, C42A (leading to a premature STOP codon), and a “G” insertion between nucleotides 296–297 or 391–392. Of note, the *pncA* L4W mutation was common to all 12-banded isolates, confirming their resistance to pyrazinamide and clonality.

To gain insights into the evolution of drug resistance among the outbreak patients, we expanded the mutational analysis to the latest isolate of each patient whose treatment failed. Three patients were found to harbor a pre-XDR strain, one of whom evolved into XDR since its corresponding isolate successively accumulated the ofloxacin resistance mutation *gyr*A A90V and the kanamycin/amikacine resistance mutation *rrs* A1401G. The XDR status of the infecting strain was further confirmed phenotypically (data not shown).

### Clinical evolution of the MDR-TB outbreak patients and predictors of unfavorable treatment outcomes

**Fig 2:**
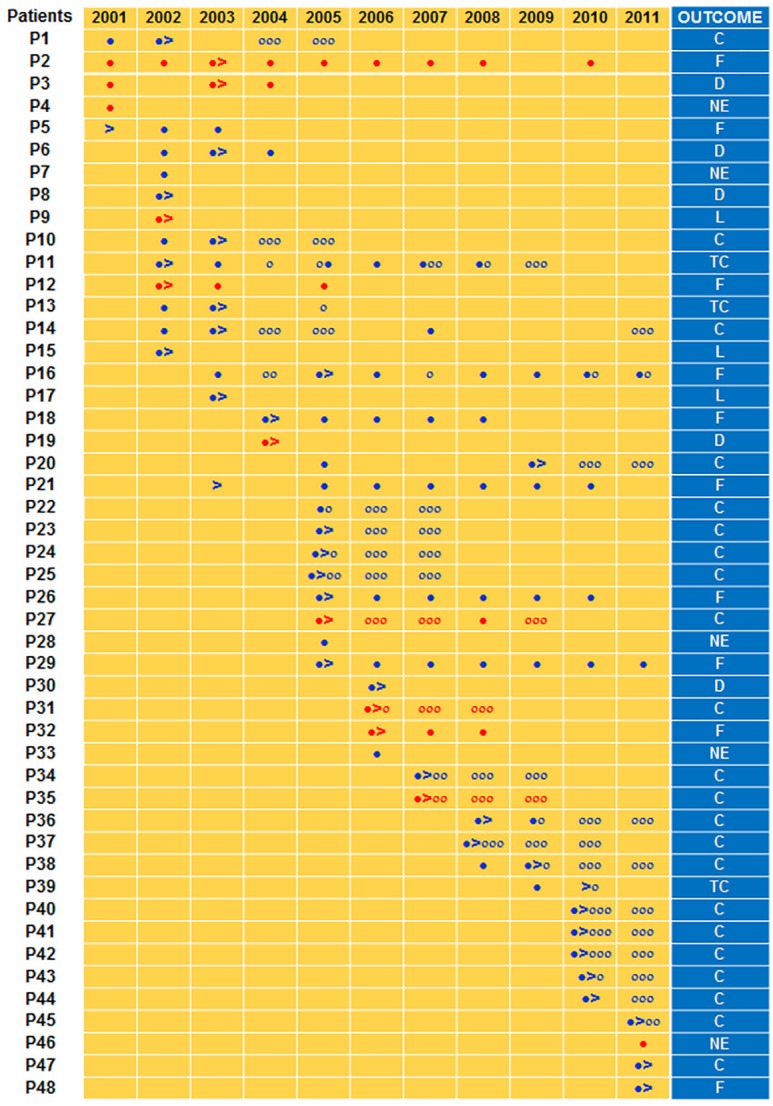
Culture results, time of initiation of second-line chemotherapy, and treatment outcomes for each patient involved in the Haarlem3 MDR-TB outbreak. Red and blue filled circles indicate a positive culture for patients with a 12-banded and 11-banded IS*6110* RFLP outbreak strain, respectively. Red and blue empty circles indicate a negative culture for patients with a 12-banded and 11-banded IS*6110* RFLP outbreak strain, respectively. Time of initiation of second line drugs uptake is indicated by >. Treatment outcomes were based on the WHO definitions and reporting framework for tuberculosis that was revised in 2013 and updated in December 2014 [2]. C: cured; TC: treatment completed; F: failed; D: died; L: lost to follow-up; NE: not evaluated

Of the 48 Haarlem3 MDR-TB outbreak patients, 22 (45.8%) were cured, 3 (6.2%) completed treatment, 10 (20.8%) failed treatment, 3 (6.2%) were lost to follow-up, 5 (10.4%) died, and 5 (10.4%) could not be evaluated. Hence, the overall treatment success rate (cured + treatment completed) for this MDR-TB outbreak was 52.1%. The results of culture, date of initiation of second-line chemotherapy and treatment outcomes for each patient involved in the Haarlem3 MDR-TB outbreak are shown in details in Fig 2.

**Fig 3:**
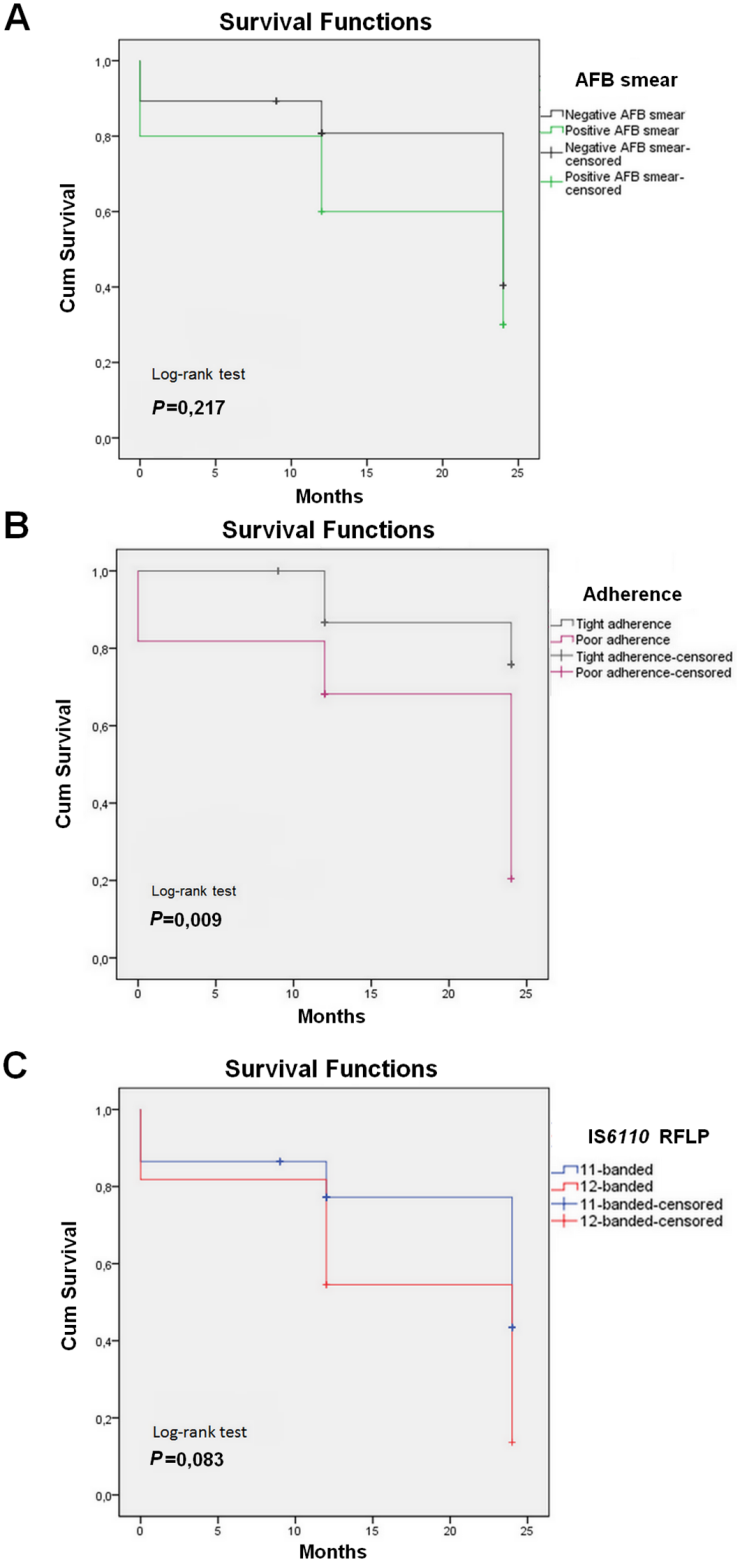
Kaplan–Meier plots and log-rank test for probability of unfavorable outcomes, according to AFP smear positivity (A), poor adherence to treatment (B), and IS*6110* RFLP pattern of the outbreak strain (C).

There were no differences in treatment outcomes between patients regarding age, sex, smoking history, links with known MDR cases, chest radiography, pyrazinamide resistance of the infecting strain, underlying diseases, prior DOTS chemotherapy, or incarceration (Table 2). However, patients with positive AFB smear and those who poorly adhered to second-line chemotherapy (irregular uptake of drugs and/or interruptions) were more likely to evolve unfavorably (P = 0.023 and *P*<0.04, respectively) (Table 2), a finding in line with Kaplan-Meier analyses (Fig 3A and 3B, respectively).

**Table 2:** Clinical outcomes for the 41 Haarlem3 MDR-TB outbreak patients that had received a 2-year second line anti-TB chemotherapy.

	All outcomes (n = 41)	Treatment success (n = 24)	Unsuccessful treatment outcomes (n = 17)	*P* value
**Age (mean)**	31.15 (12.764)	31.17 (12.139)	31.12 (13.982)	0.991
<30	25 (61.0%)	14 (58.3%)	11 (64.7%)	
30–50	11 (26.8%)	05 (20.8%)	6 (35.3%)	0.112
>50	05 (12.2%)	05 (20.8%)	0 (0.0%)	
**Male**	36 (87,8%)	19 (79.2%)	17 (100%)	0.065
**Smoking history**	28 (68.3%)	14 (58.3%)	14 (82,4%)	0.173
**Employed**	25 (61%)	15 (62.5%)	10 (58.8%)	1.000
**Epidemiological link with MDR cases**	11 (26.8%)	5 (20.8%)	6 (35.3%)	0.476
Positive AFB smear prior to SLD*	17 (41.5%)	6 (25.0%)	11 (64.7%)	**0.023**
Pre-SLD* Finding of CXR				
Cavitary lesions	6 (14.6%)	3 (12.5%)	3 (17.6%)	0.679
Pleural effusion	4 (9.8%)	3 (12.5%)	1 (5.9%)	0.629
Bilateral lesions	16 (39.0%)	8 (33.3%)	8 (47.1%)	0.518
Unilateral	15 (36.6%)	10 (41.7%)	5 (29.4%)	0.679
**Comorbidities**				
Diabetes	4 (9.8%)	2 (8.3%)	2 (11.8%)	1.000
Respiratory diseases other than TB	6 (14.6%)	4 (16.7%)	2 (11.8%)	1.000
Cancer	5 (12.2%)	2 (8.3%)	3 (17.6%)	0.633
Others	12 (29.3%)	6 (25.0%)	6 (35.3%)	0.507
**Resistance to pyrazinamide**	29 (70.7%)	15 (62.5%)	14 (82.4%)	0.296
**IS*6110* RFLP profile**				
11-banded	31 (75.6%)	21 (87.5%)	10 (58.8%)	**0.063**
12-banded	10 (24.4%)	3 (12.5%)	7 (41.2%)	
**Previous or present incarceration**	12 (29.3%)	6 (25.5%)	6 (35.3%)	0.507
**Prior DOTS treatment**	29 (70.7%)	15 (62.5%)	14 (82.4%)	0.296
**Poor adherence to treatment**	22 (53.7%)	8 (33.3%)	14 (82.4%)	**0.004**

*SLD: Second-line drugs.

Significant *P* values are shown in bold.

Intriguingly, patients that had been infected with the 12-banded outbreak strain tended to evolve unfavorably (*P* = 0.063) (Table 2). This was further confirmed by Kaplan-Meier analyses, which yielded a borderline statistically significant association (*P* = 0.083) of the 12-banded-infected patients and a more rapid evolution towards an unfavorable outcome (Fig 3C).

Univariate analysis for comparison of patients with successful and unsuccessful treatment outcomes revealed that infection with a 12-banded outbreak strain (HR: 4.90; 95% CI 1.04–23.04, *P* = 0.044), positive AFB smear (HR: 5.50; 95% CI 1.41–21.39; *P* = 0.014), and poor adherence to treatment (HR: 9.33; 95% CI 2.06–42.18; *P* = 0.004) were all significantly associated with unfavorable outcomes (Table 3). Of these clinical variables, the latter proved to be the main independent predictor (HR: 9.15; 95% CI 1.72–48.73; *P* = 0.014) of unfavorable outcomes in the final multiple logistic regression model (Table 3).

**Table 3:** Predictors of unfavorable outcomes for the MDR-TB outbreak patients that had received a 2-year second line anti-TB chemotherapy.

	Univariate Analysis				Multivariate logistic regression			
	HR	95% CI		*P* value	HR	95% CI		*P* value
**Infection with a 12-banded strain**	4.90	1.04	23.04	**0.044**	2.94	0.46	18.730	0.254
Positive AFB smear prior to SLD*	5.50	1.41	21.39	**0.014**	4.46	0.88	22.57	0.071
**Resistance to pyrazinamide**	2.80	0.63	12.49	0.177	-	-	-	-
**Poor adherence to treatment**	9.33	2.06	42.18	**0.004**	9.15	1.72	48.73	**0.009**
**Prior DOTS treatment**	2.80	0.63	12.49	0.177	-	-	-	-
**Previous or present incarceration**	1.63	0.42	6.36	0.477	-	-	-	-
**Employed**	0.85	0.24	3.06	0.812	-	-	-	-
**Smoking history**	3.33	0.75	14.75	0.113	-	-	-	-
**Comorbidities**	0.75	0.22	2.61	0.654	-	-	-	-
**Epidemiological link with MDR cases**	2.07	0.51	8.40	0.308	-	-	-	-

*SLD: Second-line drugs

HR: Hazard ratio

CI: confidence interval

Significant *P* values are indicated in bold.

### Clinical evolution of the Haarlem3 MDR-TB outbreak patients throughout the whole study period

To appreciate the evolution of the Haarlem3 MDR-TB outbreak at the long-term, we assessed the clinical outcomes of patients for several years (2 to 8 years) beyond the 24-month period of second-line treatment. It came out that among the 10 patients whose treatment failed, 7 (70%) have died, 1 (10%) was cured, and 2 (20%) are still under treatment. Of the 25 successfully treated patients, 6 (24%) have relapsed.

## Discussion

Here we described in details the evolution of an MDR-TB outbreak and the treatment outcomes for the involved patients over an 11-year period. This outbreak emerged and successfully expanded in a context strictly negative for HIV infection, within a country whose HIV/AIDS prevalence is very low (<0.1%)[20], thus challenging the long-standing belief that MDR-TB strains are transmission-deficient and preferentially expand among immunocompromised patients, as opposed to immunocompetent individuals [3,8–10]. In a well documented case that occurred in Argentina, the spread of an MDR-TB outbreak among HIV-negative individuals was shown to be the extension of a transmission chain that first involved HIV-positive hospitalized patients [21–23]. This appears to be the case for most MDR-TB outbreaks that expanded among HIV-negative individuals, particularly in countries with high burden of HIV infection [24]. Therefore, describing the evolution of the MDR-TB outbreak that occurred in Northern Tunisia does not suffer from the major confounding effect of HIV infection.

The MDR-TB outbreak described herein is due to a strain of the Haarlem genotype, which is the most predominant genotype in the north-east of Tunisia, and which is characterized by higher recent transmission rates over other prevalent genotypes, particularly the Latin American Mediterranean (LAM) and T genotypes [25]. Therefore, the successful transmission of this MDR-TB outbreak would have benefited of the intrinsic ability of the Haarlem genotype to undergo rapid transmission in this particular region.

**Fig 4:**
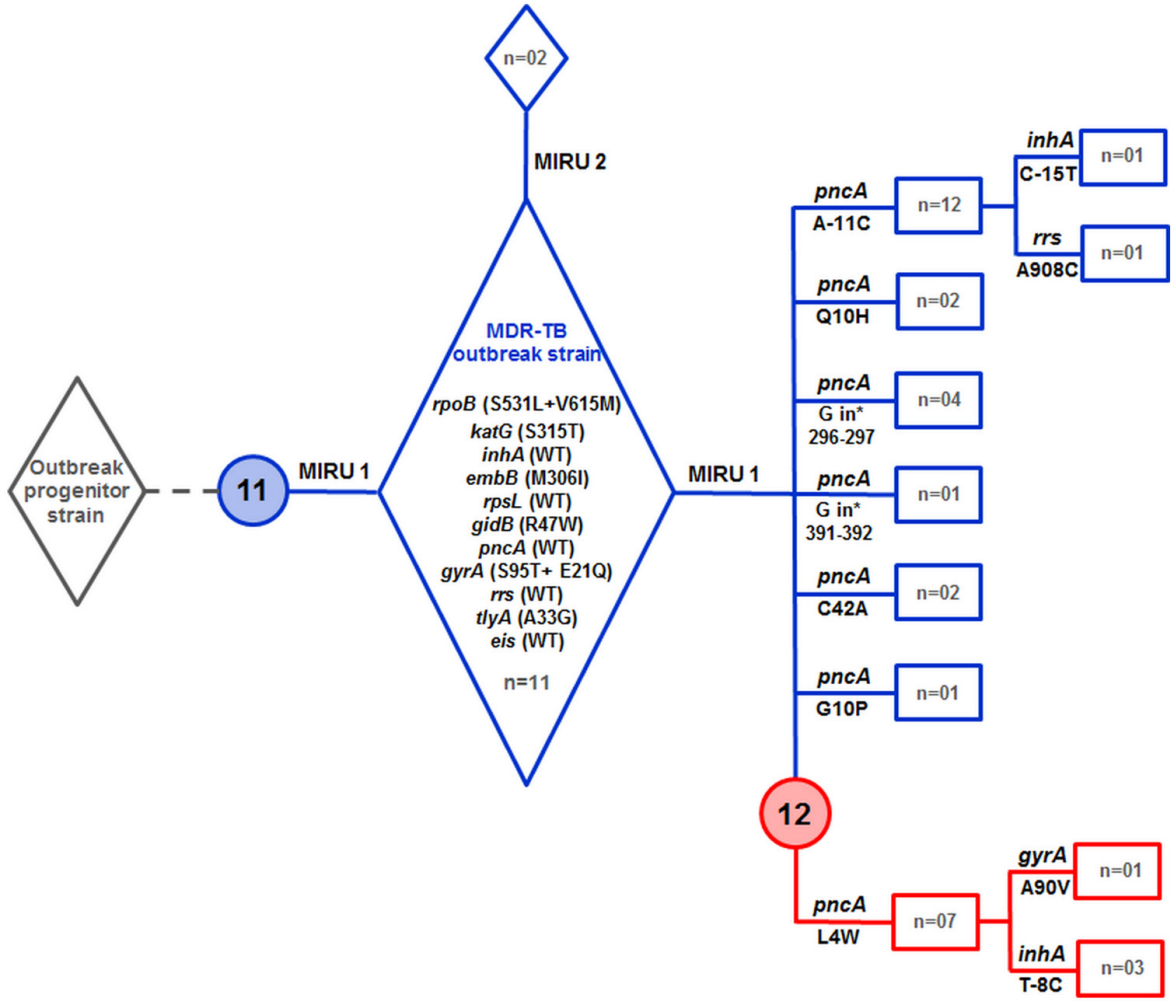
Emergence and expansion scenario of the Tunisian Haarlem3 MDR-TB outbreak based on genotypic analyses and accumulation of mutations in drug resistance genes. Numbers 11 and 12 within the blue and red circles indicate the 11-banded and 12-banded RFLP profiles, respectively. Lanes in blue indicate outbreak strains that evolved as 11-banded IS*6110* RFLP profile, while lanes in red indicate those with the 12-banded IS*6110* RFLP profile. The number of strains (n =) with a particular genotypic and drug resistance mutation profile is indicated. The detailed MIRU-VNTR24 patterns of the outbreak strains (MIRU1 and MIRU2) are shown in S1 Table.

Genotypic and mutational analysis of drug resistance genes allowed us to reconstruct a plausible emergence and expansion scenario for the MDR-TB outbreak. As shown in Fig 4, the outbreak strain emerged from the pansusceptible progenitor by accumulating several drug resistance-conferring mutations, leading to the MDR phenotype. The fact that we have not been able to identify monodrug-resistant isolates that are genetically closely related to the MDR-TB outbreak strain suggests that acquisition of multiple mutations in drug resistance genes was a rapid process.

Conversion to the MDR phenotype of the progenitor strain seemed to have been accompanied, from the outset, with the acquisition of the epidemic phenotype, since at least 13 patients were identified whose isolates showed identical mutations in *rpoB*, *katG*, *embB*, *gidB*, *tlyA*, and *gyrA*. Recently, we showed that the secondary *rpoB* mutation, V615M, fully restored the fitness defect incurred by the rifampicin resistance mutation S531L [26]. The finding that *rpoB* V615M and *rpoB* S531L occurred concomitantly argues for a streamlined fitness costs compensation process, which in turn could have significantly contributed to the epidemic potential of this Haarlem3 MDR-TB outbreak strain.

Several lines of evidence suggest that the outbreak strain first emerged as an 11-banded RFLP profile. Indeed, this profile was predominant among the outbreak patients and included strains that were still susceptible to pyrazinamide. Moreover, patient P14, whose strain displayed the 11-banded profile, might be the index case since he presented to the hospital as early as 1997, before any other patient known to be linked to the Haarlem3 MDR-TB outbreak. Of note, this patient had been receiving first-line drugs for 5 years before DST revealed the MDR nature of his strain. Therefore, it is very likely that patient P14 contributed significantly to the initial wave of MDR-TB transmission, inasmuch as contact tracing identified three patients that were linked to him. Because the double mutation *rpoB* S531L/*rpoB* V615M was shown to confer high-level resistance to rifampicin (MIC>160) [26], the most powerful anti-tubercular drug, one can reasonably argue that the use of first-line drugs, early at the onset of the MDR-TB outbreak, should not have contributed in reducing the bacillary load in patients’ expectorates.

As the MDR-TB outbreak strain expanded, it developed resistance to pyrazinamide. As a matter of fact, before undergoing second-line drugs chemotherapy, most of the outbreak patients had been receiving a standard first-line drug regimen, while their strains were simultaneously resistant to isoniazid, rifampicin, and ethambutol. Patients were thus on a functional pyrazinamide monotherapy, whose immediate consequence was acquisition of resistance to pyrazinamide. The heterogeneous pattern of mutations in the *pncA* gene, suggests independent acquisition of resistance to pyrazinamide, and confirms the results of previous studies reporting high diversity of *pncA* mutations within large collections of pyrazinamide-resistant isolates [27–29]. However, the *pncA* mutations A-11C and L4W involved 14 and 11patients, respectively, while the other mutations were associated with 1 to 4 cases only. Given the clonal nature of the outbreak strains, it is therefore tempting to speculate that different *pncA* mutations could have variable effects on fitness, a suggestion in line with a recent study proposing that pyrazinamide resistance typically induces a fitness cost that impairs *M*. *tuberculosis* transmission [30]. The *pncA* mutation L4W is of particular interest since it is concomitant with the emergence of a new outbreak subclone characterized by an additional RFLP copy. Instability of IS*6110* in clusters of MDR-TB strains has been previously reported [31,32], and thus confirmed herein.

Additional mutations in drug resistance-conferring genes resulted in three pre-XDR strains, from which one XDR strain evolved by 2010. Hence, if we consider that the MDR phenotype could have been acquired between years 1997 and 2000, then it took about one decade of evolution for the outbreak to evolve one XDR strain. In the case of the major MDR-TB outbreak that took place in Argentina, it was estimated that evolution to the XDR phenotype took about 18 years after the strain became MDR [23]. These two naturally occurring situations show that evolution of MDR-TB outbreak strains to the XDR phenotype takes several years, leaving a precious time for health authorities to contain the ongoing transmission, and thus prevent the emergence of highly transmissible XDR-TB strains. However, the time to evolve to XDR may be determined by the strain genotype. Indeed, it has been shown that *M*. *tuberculosis* strains from lineage 2 (East Asian lineage and Beijing sublineage) acquire drug resistances *in vitro* more rapidly than strains from lineage 4 (Euro-American lineage). This finding was attributed to a higher mutation rate of lineage 2 strains, which correlated well with the bacterial mutation rate of clinical *M*. *tuberculosis* isolates in humans [33]. It remains to be seen, whether the mutation rate of the lineage 4 Haarlem3 outbreak strain described herein could have influenced its evolution to the XDR phenotype.

The overall treatment success rate of 52.1% estimated for this Haarlem3 MDR-TB outbreak is very close to the 51.8% success rate reported for the MDR-TB outbreak of Argentina [21,34]. Curiously, both outbreaks are due to a Haarlem genotype strain. However, these treatment success rates are below the overall 62–65%success rates estimated from meta-analyses [35,36], and far below the treatment success rate of 81% achieved for HIV-negative MDR-TB patients in Ethiopia [37]. Hence, it seems likely that during outbreaks, rapid transmission of MDR-TB strains may complicate the management of cases, thus resulting in lower treatment success rates. The finding that the evolved 12-banded subclone was significantly associated with unfavorable outcomes, suggests that the outbreak strain could have evolved towards increased fitness as transmission progressed.

Our multivariate analysis revealed that poor adherence to treatment is predictive of unfavorable outcomes. Therefore, understanding the factors that contribute to non-compliance with drug treatments will be critical to achieving higher success rates in this particular MDR-TB outbreak. The unfavorable fate of patients observed several years after the 24-month treatment period, should call the health authorities to consider extending the follow-up of outbreak patients for a long time beyond the treatment period. Furthermore, early detection of new MDR-TB cases using newly developed nucleic acid-based approaches should be systematically applied for individuals with a suspected link to the outbreak patients. Also to help preventing expansion of this Haarlem3 MDR-TB outbreak, active case detection among the outbreak patients’ contacts must be undertaken.

Based on previous findings, and taking into account the data obtained herein, it is reasonable to argue that the successful expansion of the Haarlem3 MDR-TB outbreak could have resulted from factors related to the involved strain, and from the consequences of delayed identification of MDR-TB cases. Indeed, the causing Haarlem3 strain belongs to a predominant and rapidly transmitted genotype, and could thus be endowed with an intrinsic epidemic phenotype [6,25]. In addition, we recently provided strong evidence that this Haarlem3 outbreak strain has undergone a compensatory evolution, mediated by a secondary site *rpoB* mutation, *rpoB* V615M, which not only preserved its overall fitness, but also significantly increased its resistance level to rifampicin, one of the most potent anti-tubercular drug [26]. Furthermore, because of strong delays in DST results, MDR-TB patients had been receiving first-line chemotherapy long before switching to second-line treatment, a fact that has very likely contributed to increased transmission and acquisition of additional resistance, notably to pyrazinamide. Therefore, detecting MDR-TB cases at early stages is of uppermost importance and must be the main objective of the NTP program in this particular outbreak region. Finally, early identification of MDR-TB cases with immediate administration of second-line drugs will certainly help reducing the rate of nonadherence, which was shown here to be significantly associated with unfavorable outcomes, and hence perpetuation of the MDR-TB outbreak.

## Conclusions

We have described, over an 11-year period, an MDR-TB outbreak that evolved in an HIV-negative context, Northern Tunisia. We showed that the involved MDR-TB strain proved fit enough to cause a large outbreak among HIV-negative young individuals, and tended to evolve towards increased severity. The observed low rates of cure and high rates of fatality and relapse prompt to raise the bar of surveillance of MDR-TB outbreaks that particularly evolve in HIV-negative settings.

## Supporting Information

S1 Table Genotypic and mutational analysis of drug resistance-conferring genes of the 48 MDR-TB outbreak strains. *Drug susceptibility results for the following anti-tubercular drugs: Isoniazid, streptomycin, rifampicin, ethambutol, and pyrazinamide. R: resistant; S: Susceptible
(XLSX)

S2 Table Characteristics of primers used to detect resistance mutations to first-line and second-line anti-tubercular drug. (DOCX)
