# Supplementary material for: MDR-TB Outbreak among HIV-Negative Tunisian Patients followed during 11 Years
Source: PLoS One. 2016 Apr 28;11(4):e0153983. doi: 10.1371/journal.pone.0153983 (PMC4849785; doi:10.1371/journal.pone.0153983)
Supplement: S2 Table — (DOCX) [file pone.0153983.s002.docx]

**S2 Table.** Characteristics of primers used to detect resistance mutations to first-line and second-line anti-tubercular drug.

| **Primer designation** | **Sequence (5’ to 3’)** | **Lenght (bp)** | **Sense** | **Position** | **Amplicon size (bp)** |
| --- | --- | --- | --- | --- | --- |
| rpoB-1f | ATCACACCGCAGACGTTG | 18 | F | 1216-1233 | 752 |
| rpoB-2r | TGCATCACAGTGATGTAGTCG | 21 | R | 1947-1967 | 752 |
| inhA-1f | TGCCCAGAAAGGGATCCGTCATG | 23 | F | (-162)-(-140) | 455 |
| inhA-2r | ATGAGGAATGCGTCCGCGGA | 20 | R | 436-455 | 455 |
| katG-1f | AACGACGTCGAAACAGCGGC | 20 | F | 1433-1452 | 455 |
| katG-2r | GCGAACTCGTCGGCCAATTC | 20 | R | 998-1017 | 455 |
| gyrA-1f | CCCTGCGTTCGATTGCAAAC | 20 | F | (-29)-(-9) | 423 |
| gyrA-2r | CTTCGGTGTACCTCATCGCC | 20 | R | 375-394 | 423 |
| rrs-1f | GTCAACTCGGAGGAAGGTGG | 20 | F | 1158-1177 | 516 |
| rrs-2r | GTCCGAGTGTTGCCTCAGG | 19 | R | (+118)-(+136) | 516 |
| eis-1f | GCGTAACGTCACGGCGAAATTC | 22 | F | (-124)-(-145) | 567 |
| eis-2r | GTCAGCTCATGCAAGGTG | 18 | R | 404-421 | 567 |
| tlyA-1f | AGGCGCACGAGGTGTTGTTG | 20 | F | (-57)-(-38) | 528 |
| tlyA-2r | AACGACAGGTCGGCCACTACCAGGT | 25 | R | 446-470 | 528 |
| tlyA-3f | ATGTCGGATACGGCCAGCTG | 20 | F | 334-353 | 555 |
| tlyA-4r | ACTTTTTCTACGCGCCGTGC | 20 | R | (+62)-(+81) | 555 |
| embB-1f | CTGACCGACGCCGTGGTGATAT | 22 | F | 834-855 | 490 |
| embB-2r | TGAATGCGGCGGTAACGACG | 20 | R | 1305-1324 | 490 |
| pncA-1f | GGCTGCCGCGTCGGTAGG | 18 | F | (-44)-(-26) | 640 |
| pncA-2r | GCCGCCAACAGTTCATCCC | 19 | R | 577-596 | 640 |
| rpsL-1f | CGTGAAAGCGCCCAAGATAG | 20 | F | (-30)-(+10) | 333 |
| rpsL-2r | GAACCGCGGATGATCTTGTAG | 21 | R | 282-303 | 333 |

F: forward

R: reverse
